# Bronchial artery diameter in massive hemoptysis in cystic fibrosis

**DOI:** 10.1186/s12890-022-02233-2

**Published:** 2022-11-17

**Authors:** Martha Dohna, Hilmar Kühl, Sivagurunathan Sutharsan, Christian Dohna-Schwake, Van Dai Vo Chieu, Susanne Hellms, Norman Kornemann, Diane M. Renz, Michael J. Montag

**Affiliations:** 1 Department of Pediatric Radiology, Hannover Medical School, Institute of Diagnostic and Interventional Radiology, Carl-Neuberg-Str. 1, 30625 Hannover, Germany; 2 St. Bernhard-Hospital Kamp-Lintfort, Bürgermeister-Schmelzing-Str. 90, 47475 Kamp-Lintfort, Germany; 3 Division of Cystic Fibrosis, Department of Pulmonary Medicine, University Medicine Essen -Ruhrlandklinik, University of Duisburg-Essen, Essen, Germany; 4 Department of Pediatrics I, University Medicine Essen, Hufelandstr 55, 45147 Essen, Germany; 5 St. Vicenz-Hospital Paderborn, Am Busdorf 2, 33098 Paderborn, Germany; 6 Clinic for Radiology, University Hospital Münster, Albert-Schweitzer-Campus 1, 48149 Münster, Germany

**Keywords:** Cystic fibrosis, Massive hemoptysis, Bronchial artery diameter, Risk factor

## Abstract

**Background:**

Massive hemoptysis is a rare but potentially life-threatening condition of patients with cystic fibrosis (CF) and advanced pulmonary disease. Hypertrophied bronchial arteries are understood to cause massive hemoptysis when rupturing. Risk factors to predict massive hemoptysis are scarce and bronchial artery diameters are not part of any scoring system in follow-up of patients with CF. Aim of this study was to correlate bronchial artery diameter with massive hemoptysis in CF.

**Methods:**

Bronchial artery and non-bronchial systemic artery diameters were measured in contrast enhanced computed tomography (CT) scans in patients with massive hemoptysis and compared to patients with end-stage CF and no history of hemoptysis. Demographic and clinical data and side of bronchial artery/non-bronchial systemic artery hypertrophy and coil embolization were documented.

**Results:**

In this retrospective multicenter study 33 patients with massive hemoptysis were included for bronchial artery/non-bronchial systemic artery diameter measurements, (13 female, 20 male, median age 30 years (18–55)). Bronchial artery diameters were significantly larger in the case group than in the control group with median 4 mm (2.2–8.2 mm), and median 3 mm (1–7 mm), respectively (*p* = 0.002). Sensitivity of bronchial arteries ≥ 3.5 mm to be associated with hemoptysis was 0.76 and specificity 0.71 with ROC creating an area under the curve of 0.719. If non-bronchial systemic arteries were present, they were considered culprit and embolized in 92% of cases.

**Conclusion:**

Bronchial arteries ≥ 3.5 mm and presence of hypertrophied non-bronchial systemic arteries correlate with massive hemoptysis in patients with CF and might serve as risk predictor for massive hemoptysis. Therefore, in patients with advanced CF we propose CT scans to be carried out as CT angiography to search for bronchial arteries ≥ 3.5 mm and for hypertrophied non-bronchial systemic arteries as possible risk factors for massive hemoptysis.

## Background

Bloody stained sputum is a common finding in patients with cystic fibrosis (CF) [1] and in the majority of cases minor and self-limiting. But mild, and especially recurrent minor hemoptysis might be a warning of impending massive hemoptysis (MH) [2]. MH is a potentially life-threatening condition which predominantly occurs in advanced CF with mortality rates as high as 75% [3]. 1–4.1% of patients with CF will experience MH with an annual incidence of approximately 1%. In many cases of MH immediate intervention is necessary, often carried out in an emergency setting [4, 5]. If hemoptysis is major but does not require immediate intervention patients might be listed for lung transplant. However, data on risk factors for MH in CF are scarce [2]. It is therefore crucial to identify and differentiate patients who will develop MH from those who will not and to understand and target predictors of MH.

Chronic local and systemic inflammation is understood to result in a variety of changes of lung tissue. Chronic inflammation in CF causes upregulation of serum vascular endothelial growth factor which induces hypertrophy of bronchial arteries (BA) and non-bronchial systemic arteries (NBSA). These present as enlarged, tortuous, and dilated vessels. BA are localized submucosally in the bronchial wall. Localized destruction of the airway epithelium weakens the vessel wall [4, 6]. This combination of dilated hypertrophic vessels and damaged vulnerable vessel walls might then result in rupture and massive bleeding into the airways [4, 7].

Surveillance of patients with CF includes evaluation of specific pulmonary changes and their progression over time. Typical morphologic lung tissue changes can be assessed with chest x-ray, computed tomography scans (CT), generally non-contrast-enhanced, and increasingly with magnetic resonance imaging (MRI), as protocols and imaging quality are improving [8]. Medical imaging is evaluated for morphologic lung tissue changes using different scoring systems e. g. the Helbich score [8, 9], but quantitative and qualitative assessment of BA diameters and search for non-bronchial systemic arteries (NBSA) is not part of protocols.

Aim of this study was to quantify BA hypertrophy in patients with CF and MH compared to patients with end stage CF and no history of hemoptysis to evaluate BA/NBSA diameter as possible predictors of MH.

## Material and methods

Patients or public were not involved in the design, or conduct, or reporting, or dissemination plans of our research.

### Case group

All patients with CF and MH between 12/2005 and 2/2021 who received a CT-angiography (CTA) in the context of their MH were included into this retrospective multicenter study. MH was defined as > 240 ml in 24 h or recurrent bleeding of substantial volume (> 100 ml/d) for a few days or weeks [10]. Presence of MH, BAE, listing for lung transplant, and demographic and clinical data of patients were documented.

If patients received more than one BAE, CT scans and DSA refer to the first intervention. Localization of the culprit lobe(s) was based on patient’s perception, CT scan and DSA findings and considered correct if hemostasis was obtained for at least 48 h after BAE [6, 11, 12]. Maximum diameters of BA and NBSA supplying lung tissue were measured in mm in contrast enhanced CT scans. In patients with BAE BA diameters were additionally measured in DSA as proof of principle of BA measurement in CT. CT scans and DSA were evaluated twice with a six month delay between measurements. Radiologists blinded for first measurements. Measurements were carried out in consensus by two radiologists with 8 and 35 years of experience.

For analysis of side difference of BA diameters in patients who underwent BAE for MH measurements of BA were carried out separately for right and left lung. Matching of largest BA diameter and side of MH is given in % for the case group patients who underwent ssBACE and DSA. Analysis of maximum BA diameter comparing case group with control group is based on maximum diameter of each patient independent of BA or NBSA or lateralisation. In lack of a scoring system and little to no scientific data on BA diameters we applied the scoring system used in our department with diameters < 2 mm considered physiologic as previously described in the literature (see Table 1) [13]. This scoring system is based on a large variety of pulmonary diseases with hemoptysis with patients admitted for endovascular embolization.

**Table 1:** Staging of bronchial artery and non-bronchial systemic artery diameters

Stage	Bronchial/non-bronchial systemic artery diameter
Stage 1, normal	< 2 mm
Stage 2, moderate hypertrophy	2–4 mm
Stage 3, major hypertrophy	≥ 4 mm

### Control group

We retrospectively evaluated 35 CT-A (Dual-Source-CT-Scanner SOMATOM Force, Siemens) carried out between 6/2010 and 6/2020 of patients with end stage CF aged ≥ 16 years and no history of hemoptysis to measure maximum diameter of BA. CTA had been carried out to evaluate suitability for lung transplant in 33 patients and for liver transplant evaluation in two patients. Demographic and clinical data of patients were documented. Observation interval after CT scan was 2 to 10 years (until lung transplant, death or follow-up until 8/2022).

Largest BA, and, if present, largest NBSA diameter were documented in CTA without lateralization. Enlarged vascular structures within extrapleural fat in association with pleural thickening (3 mm) were regarded as NBSA causing hemoptysis as previously described in the literature [14–17].

### Statistics

Data are presented as median giving minimum and maximum values. We used the Man-Whitney-U test to compare wo independent groups. Receiver operating characteristics (ROC) curve was calculated for BA diameters and area under the curve is given. Spearman rank correlation coefficient between CTA and DSA measurements, median values and binary logistic regression analysis for dependent variables age, sex, FEV1% pred. and BA diameter were calculated using IBM SPSS Statistics Version 27. BA diameter was correlated with age with Spearman rank correlation. Differences in BA diameters in relation to sex were calculated by Kruskal–Wallis Test. *p* < 0.05 was considered statistically significant.

## Results

Measurement for bronchial artery and non-bronchial systemic artery diameters:

### Case group

33 patients were included for BA/NBSA diameter measurements, (13 female, 20 male, median age 30 years (18–55). Median FEV1% pred. was 44 (16–104). Median BMI was 20.8 (16–28.2). Depending on the medical center patients were either listed for lung transplant (*n* = 11) or underwent BA embolization (BAE) (*n* = 22). Patients who underwent BAE for MH also received DSA. Of these 22 patients 19 received a super selective bronchial artery coil embolization (ssBACE). 19 patients were included for comparison of right and 18 patients of left lung BA diameters in DSA and CT, as in three patients DSA images were not available and in one patient angiography was only carried out for the right lung. In 16 of 19 patients ssBACE was unilateral, in 13 cases (81%) affecting only the right lung. In 13 patients BA of the right lung were larger with ssBACE carried on the right lung (100% correct). In one case BA diameters showed no side difference with ssBACE carried out only in the right lung. In three cases ssBACE was carried out on the left lung with BA diameters larger in the left lung in only one patient (33% correct). In three cases bilateral ssBACE was carried out with larger BA in the right lung in all cases.

BA showed diameters of median 4 mm (2.2–8.2 mm). Evaluation of contrast enhanced CT scans showed 17 of 19 patients (90%), and angiography 16 of 18 patients (89%) to have larger BA on the right side. BA diameters were significantly larger in the right lung (*p* < 0.001). DSA showed maximum BA diameters of 3.9 mm (2.5–6.4 mm) of right and 2.9 mm (1.8–4.2 mm) of left lung compared to CT measurements with 4 mm (2.4–6 mm) of right lung compared and 2.85 mm (1.7–4.2 mm) of left lung. CT and DSA measurements correlated very well (Spearman correlation coefficient was 0.92 (*p* < 0.001)).

**Fig. 1:**
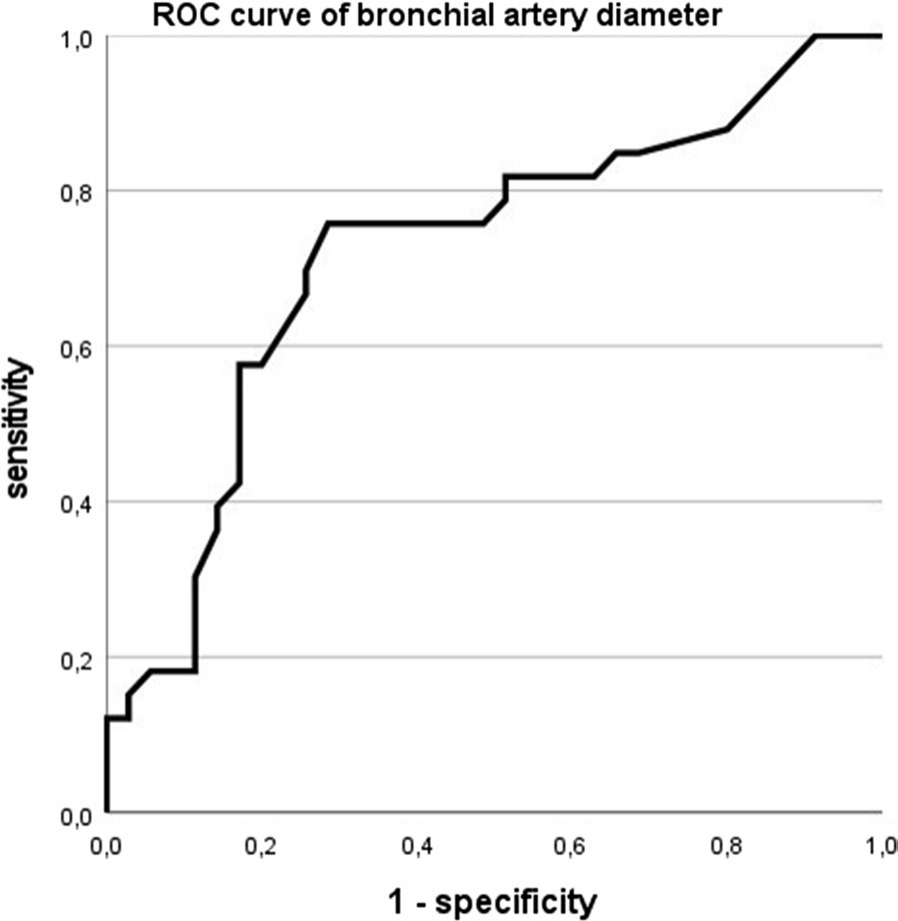
ROC = receiver operating characteristics of bronchial artery diameter and massive hemoptysis, BA = bronchial artery

CT measurements of maximum BA diameter of patients revealed 0 patients to present with BA stage 1, 15 patients to have BA stage 2 (45%), 18 patients to show BA stage 3 (55%). 25 patients (76%) showed BA ≥ 3.5 mm (see Fig. 1). BA diameters were significantly smaller in females than in males with median 3.2 mm (1.9–5.9) and 3.9 mm (2.0–8.2 mm), respectively (*p* = 0.001). BA diameter augmented with age with Spearman correlation coefficient of 0.379 (*p* = 0.001). NBSA were detected in 12 patients (36%), in ten patients right-sided and in two bilateral with diameters of median 3.2 mm (1.9–4.6 mm). Only in one patient NBSA showed a larger diameter than the BA measured in the same patient, but this did not lead to upstaging of BA diameter. In the patients undergoing BAE NBSA were embolized and considered (also) culprit in 11 of 12 cases (92%).

### Control group

35 patients were included, 16 female and 19 male, median age was 27 (15–53) years. Median FEV1% pred. was 27 (15–105) and median BMI was 18.3 (12–23.4). BA showed diameters of median 3 mm (1–7 mm). One patient (3%) showed BA diameter stage 1, 28 patients (80%) showed stage 2, and six patients (17%) showed stage 3. Ten patients (29%) showed BA diameters ≥ 3.5 mm. NBSA were detected in 10 patients (29%), in three cases right sided, in six cases left sided and in one case bilateral. Median NBSA diameter was 2.4 mm (1.7–3.9 mm).

**Fig. 2:**
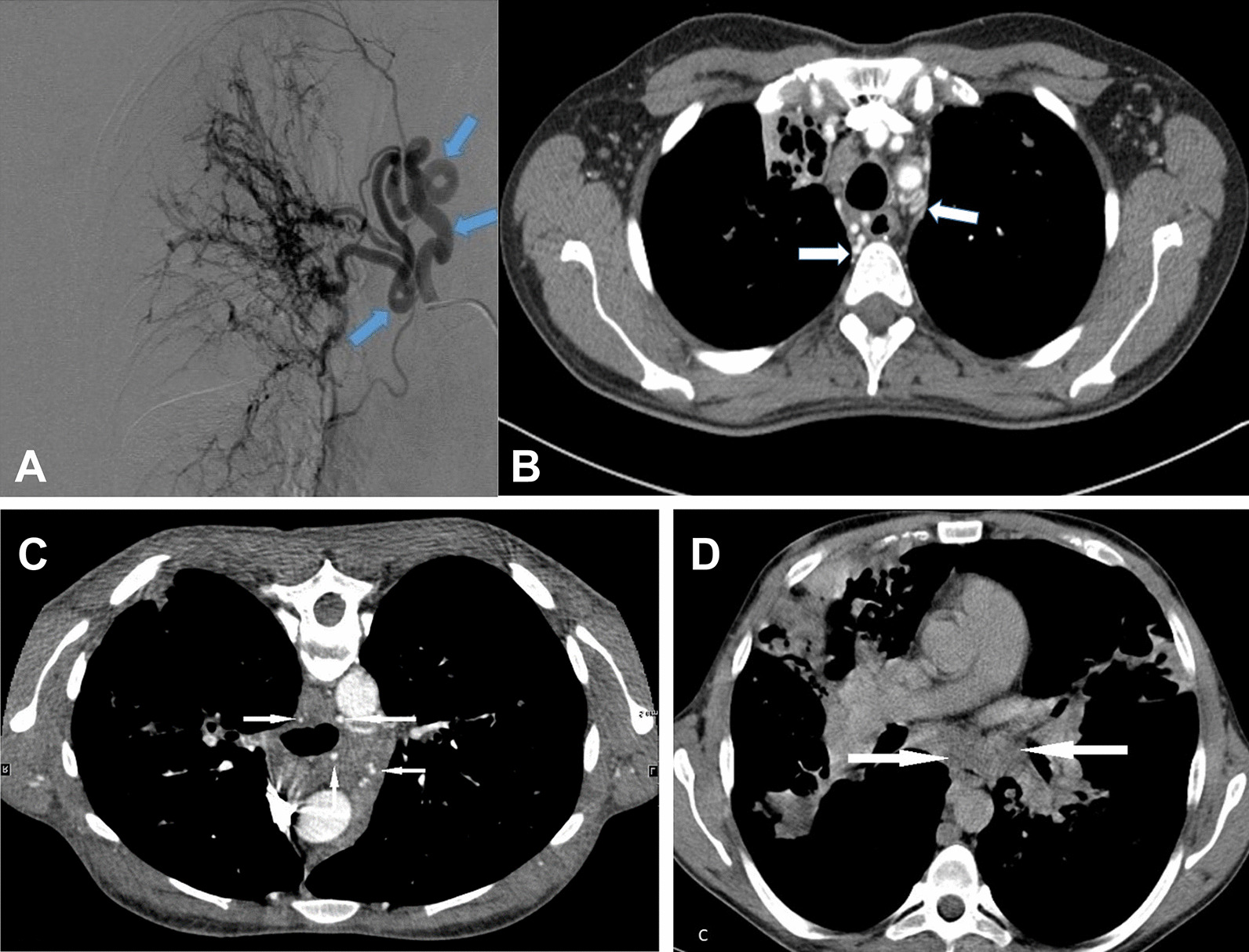
**A B**: Hypertrophy of bronchial arteries in the same patient can be seen in digital subtraction angiography (arrows in **A**) and in contrast enhanced CT scan (arrows in **B**). **C**: moderately hypertrophied bronchial arteries in a patient with end stage CF but no history of hemoptysis (arrows show bronchial artery). **D**: In this patient non-contrast-enhanced CT was performed and hypertrophied bronchial arteries cannot be distinguished from lymph nodes or other pulmonary vessels in the mediastinum (see arrows). Hypertrophy of bronchial arteries is easily missed in CT scans without contrast enhancement

Receiver operating characteristics of bronchial artery diameter and massive hemoptysis showed an area under the curve of 0.719 (see Fig. 1). BA diameters were significantly larger in the case group than in the control group (*p* = 0.002) (see Fig. 2A-C). Sensitivity of BA ≥ 3.5 mm to be associated with hemoptysis was 0.76 and specificity was 0.71. Binary logistic regression analysis with MH as dependent variable and age, sex, FEV1% pred. und BA diameter revealed BA diameter as strongest significant independent risk factor (*p* = 0.003) (see Table 2).

**Table 2:** Multivariate analysis of risk factors for MH

Risk factors for MH	Odds ratio	95% Confidence interval	significance
Bronchial artery diameter	2.932	1.45–5.95	0.003
Sex	0.587	0.16–2.1	0.42
Age	0.972	0.91–1.0	0.38
FEV1% pred	1.036	1.01–1.06	0.006

*MH* Massive hemoptysis

## Discussion

Hypertrophied BA play a pivotal role in MH and are understood to be the culprit vessels [4, 6, 7]. However, attention has never been addressed to the actual size of BA in patients with CF and MH. To our knowledge, this study is the first to correlate BA diameter with the occurrence of MH in patients with CF. MH primarily occurs in advanced CF and often creates a life-threatening emergency situation. Although chronic inflammation and lung tissue destruction is present in basically all patients with end stage CF, only few patients develop MH [1]. In addition, MH is not exclusively found in patients with advanced CF and higher age, but it also occurs in children and in patients with only minor pulmonary impairment rendering prediction of MH extremely challenging. This uncertainty leaves both patients and physicians in an unsatisfying situation when considering if and when to treat with BAE for hemoptysis [18, 19].

Despite major recent advances in treatment of CF with CFTR modulators [20], the number of patients with advanced CF and major destruction of lung tissue is high and will further increase over the next decades with rising life expectancy of patients with CF [1], most probably increasing the incidence of MH as well. Although lung transplant is a treatment option in recurrent and also MH, immediate life-saving treatment in case of MH will remain crucial [18, 19, 21]. As predictors of MH are scarce [7], their development is ever more important.

In our study BA diameters ≥ 3.5 mm were found in 76% of patients with MH (see Fig. 2A, B), whereas only in 29% of control group patients with end stage CF without hemoptysis (see Fig. 2C). However, these results might be biased as patients with bloody stained sputum and minor hemoptysis were excluded from control group in order to make differentiation of case and control group clearer. Another bias might have been created as median FEV1% pred. and also BMI of the control group are lower than in the case group. However, binary logistic regression analysis with MH as dependent variable and age, sex, FEV1 and BA diameter revealed BA diameter as the strongest independent risk factor (see Table 2). Influence of FEV1%pred. in BA diameter is minor. Control group patients had hypertrophied BA in 97%, but in the large majority (80%) only moderate hypertrophy was present. BA diameters ≥ 3.5 mm were found 2.6-fold, and BA diameters ≥ 4 mm were found 3.2-fold more often in patients with MH than in patients with end-stage CF but without history of hemoptysis. BA diameters were significantly larger in the case than in the control group creating an area under the curve of 0.719 in ROC calculation (see Fig. 1).

As BA are larger in the right lung in 90% of cases and BAE was carried out only or also on the right side in 84% of cases, we believe that measuring the largest BA in the mediastinum independent of lateralization is a feasible approach in daily routine reporting. Estimation of BA of the left lung should be considered with caution as only one of three patients showed larger BA diameters matching with MH only of the left lung. However, this number is too small for further interpretation and studies with larger numbers are necessary to create robust data for left lung BA diameter. In case of BAE special attention should be payed to patient’s perception of bleeding site as BA diameters of left lung and NBSA can be culprit vessels although they are not the largest BA/NBSA vessels. 85% of unilateral ssBACE cases were localized on the right side, which might be explained by the right lung generally being more affected by inflammatory changes than the left lung in CF [22]. BA to be larger in the more affected lung further corroborates the hypothesis of BA hypertrophy as response to chronic inflammation [4, 6].

NBSA were present in both patient and control groups. NBSA can be a major source of bleeding in 41%–88% of patients with MH [23, 24]. These prevalence numbers are also concordant with our results of 36% of patients presenting NBSA, but 92% of detected NBSA considered culprit for MH and treated. Yoon et al. described a 100% detection rate of hypertrophied BA and NBSA in CTA compared to angiography [16]. Both hypertrophied BA and NBSA could well be detected and measured in CTA with excellent correlation with measurements in DSA in the same patient. Diameters of NBSA however, did not exceed diameters of BA in the same patient and did not lead to upstaging neither in the patient nor in the control group. In addition, NBSA were found with comparable prevalence and slightly larger diameters in case group (3.2 mm (1.9 – 4.6 mm)) compared to control group 2.4 mm (1.7 – 3.9 mm). However, as 92% of detected NBSA were treated in case of MH, detection of hypertrophied NBSA is important and might serve as additional argument when considering BAE in hemoptysis.

BA diameter is not an underscore in any scoring system for CF. Routine CT scans for follow-up in patients with CF are usually carried out non-enhanced to appreciate lung parenchyma changes. Lack of contrast renders detection and measurement of BA and NBSA impossible (see Fig. 2D). In order to avoid secondary damage of ionizing radiation, morphologic lung tissue assessment is increasingly carried out as non-contrast enhanced magnetic resonance imaging (MRI) [8, 25]. BA could be detected in MRI angiography, but spacial resolution does not yet allow for BA/NBSA measurements [25]. Therefore, to carry out CT scans as CTA in patients with CF and hemoptysis might help to evaluate BA diameter and presence of hypertrophied NBSA.

Estimating the risk of future MH should be based on several risk factors and including BA diameter measurement and detection of hypertrophied NBSA in patients with CF might add another important key in individual assessment. In addition, when considering BAE it is important to remember that patients with CF report stress or anxiety and fear of bleeding in public to negatively impact their quality of life [26]. As BAE is effective and safe especially when carried out as coil embolization in treating MH, indication for BAE should also be considered in this context [6, 19, 21, 27]. In the literature several factors associated with MH have been published. MH seems to show higher prevalence with older age, FEV1% pred. < 70%, presence of diabetes and differing results for sputum colonization with *Pseudomonas aerug.* and *S. aureus* [2, 4, 6, 7, 23, 24]. Further prospective studies are necessary to validate if risk prediction of MH based on the criteria presence of NBSA and BA diameter > 3.5 mm is possible. Treatment of patients could then be safer and outcome better, as patients could be treated earlier, before occurrence of MH, in stable condition and, more importantly, not in an emergency setting.

### Limitations

Patients of control group did not receive DSA to verify BA diameters, but measurement of BA and NBSA was carried out in CTA in all patients of case and control group. In addition, comparison of DSA and CT scans was only carried out as proof of principle. BA diameter measurements in CTA and DSA to be comparable has been published in the literature [14] and showed excellent correlation in the case group. Patients with MH had CT scans with varying scanning protocols and devices but quality of CTA for detection of BA and NBSA was good to excellent in all cases. The small study group number is certainly a limitation as is the retrospective study design. However, MH is a rare complication treated in specialized centers with patients referred from all parts of Germany rendering larger cohort numbers and prospective studies difficult.

## Conclusions

Major hypertrophy of BA ≥ 3.5 mm and presence of hypertrophied NBSA correlate with MH in patients with CF and might serve as risk predictor for MH. Therefore, in patients with advanced CF we propose CT scans to be carried out as CTA to search for BA ≥ 3.5 mm and for hypertrophied NBSA to help assess the risk of possibly impending MH.

## Data Availability

The data that support the findings of this study are available from the authors of this article but restrictions apply to the availability of these data, which were used under license for the current study, and so are not publicly available. Data are however available from the authors upon reasonable request and with permission of the different institutions where patients are affiliated. Contact corresponding author MD for data request.

## Abbreviations

BA: Bronchial artery
BAE: Bronchial artery embolization
CF: Cystic Fibrosis
CT: Computed tomography
CTA: Computed tomography angiography
DSA: Digital subtraction angiography
MH: Massive hemoptysis
NBSA: Non-bronchial systemic artery
ssBACE: Super selective bronchial artery coil embolization
